# Evaluating validity of various acupuncture device types: a random sequence clinical trial

**DOI:** 10.1186/s12906-016-1026-z

**Published:** 2016-02-02

**Authors:** Jungtae Leem, Jimin Park, Gajin Han, Seulgi Eun, Meena M. Makary, Kyungmo Park, Junhee Lee, Sanghoon Lee

**Affiliations:** 1 Korean Medicine Clinical Trial Center, College of Korean Medicine, Kyung Hee University, Seoul, South Korea; 2 Department of Clinical Research of Korean Medicine, College of Korean Medicine, Kyung Hee University, Seoul, South Korea; 3 Department of Acupuncture and Moxibustion, College of Korean Medicine, Kyung Hee University, Hoegi-dong 1, Dongdaemun-gu, Seoul, 130-701 Republic of Korea; 4 Department of Gastroenterology, College of Korean Medicine, Kyung Hee University, Seoul, South Korea; 5 Department of Biomedical Engineering, Kyung Hee University, Yongin, Gyeonggi South Korea; 6 Systems and Biomedical Engineering Department, Faculty of Engineering, Cairo University, Giza, Egypt; 7 Department of Sasang Constitutional Medicine, College of Korean Medicine, Kyung Hee University, Seoul, South Korea

**Keywords:** Placebo acupuncture, Blinding, Validation, Park sham device, Streitberger’s needle, Phantom acupuncture

## Abstract

**Background:**

Although various placebo acupuncture devices have been developed and used in acupuncture research, there is controversy concerning whether these devices really serve as appropriate placebos for control groups.

**Methods/Design:**

The proposed study is a single-center prospective random sequence participant- and assessor-blinded trial with two parallel arms. A total of 76 participants will be randomly assigned to Group 1 or Group 2 in a 1:1 ratio. Group 1 will consist of Sham Streitberger’s needle, Real Streitberger’s needle, and Phantom acupuncture session. Group 2 will consist of Park Sham device with real needle, Park Sham device with sham needle, and no treatment session. Participants will have a total of three acupuncture sessions in a day. The primary endpoint is blinding test questionnaire 1. Secondary endpoints are the Bang’s blinding index, the Massachusetts General Hospital Acupuncture Sensation Scale index, and physiological data including heart rate, heart rate variability, and skin conductance response.

**Discussion:**

This trial will evaluate the relevance of using placebo acupuncture devices as controls using a validation test procedure.

**Trial registration:**

Clinical Research Information Service: KCT0001347.

## Background

Acupuncture is a treatment modality that has been used in Eastern Asia for more than 2000 years. An increased interest in the clinical effects of acupuncture has also been expressed in the West in recent years. In a 1997 report on the effects of acupuncture, the National Institutes of Health (NIH) stressed the need for evidence of efficacy [1]. Subsequently, the number of randomized controlled clinical trials (RCTs) using acupuncture to treat various conditions has rapidly increased; however, many acupuncture clinical trials have methodological limitations. Establishing appropriate placebo control groups is one of several issues. Ideally, placebo acupuncture should satisfy two conditions: (1) it should be indistinguishable from real acupuncture to blinded participants, and (2) it should be physiologically inert. Various placebo acupuncture devices that satisfy these conditions have been developed and used in acupuncture clinical trials.

Currently, there are two types of acupuncture controls: (1) sham acupuncture, in which real acupuncture needles are inserted into the skin either fully at non-acupuncture points or superficially at acupuncture points, non-acupuncture points, or non-relevant acupuncture points with certain conditions, and (2) placebo acupuncture, which uses non-penetrating acupuncture devices (i.e., blunt tip needles or non-needle devices) [2].

Various non-penetrating placebo acupuncture devices such as Streitberger’s needle, the Park sham device (PSD), and the Takakura needle have been developed. The first device, Streitberger’s needle, was developed in 1998 and used a plastic ring covered with a plastic sheet to place the needle [3]. When the practitioner taps the needle, it moves inside the handle and appears as if the acupuncture needle punctures the skin. However, it does not puncture the skin, but only a pricking sensation is felt by the patient because the tip is blunt.

In 1999, Park et al. developed the PSD which was similar to Streitberger’s needle [4]. The PSD included a flange with adhesive tape and a guide tube to place the placebo needle on the acupuncture point, while Streitberger’s needle used a plastic ring and sheet. Although these two placebo acupuncture devices were successfully applied to blinded participants and their use validated, there still remains the limitation that they induce physiological activity by touching the skin.

In 2014, Lee et al. developed a novel form of placebo acupuncture, phantom acupuncture, which induces participant expectation without any tactile stimulation such as palpation, needle insertion, or manipulation and which was also validated using blinded participants [5]. However, complex equipment is needed to perform phantom acupuncture. Therefore, it may be difficult to apply phantom acupuncture in clinical acupuncture trials.

Although various placebo acupuncture devices have been developed and used in acupuncture research, there is controversy concerning whether these devices are really appropriate for placebo control groups.

This trial will evaluate various placebo acupuncture devices that have been used in acupuncture clinical trials using a validation test to determine if they are suitable to be used in control groups.

## Methods/Design

### Objectives

The objectives of this trial are to evaluate the validity of different types of placebo acupuncture devices and determine their suitability for use as placebos in clinical trials.

### Trial design and study setting

This study will be a single center, prospective, randomized, random sequence, participant and assessor blinded trial with two parallel arms.

We will conduct the study in the Kyung Hee University Medical Center.

There are arm1 (Group1) and arm2 (Group2) in our trial that is separated. Each group has three different intervention sessions in random sequence. The order of intervention will be randomized.

### Inclusion and exclusion criteria

Participants who meet the following criteria will be included irrespective of previous acupuncture experience: (1) healthy participants 20–50 years old, and (2) participants who can communicate and complete the questionnaire.

Participants will be excluded if they meet any of the following criteria: (1) individuals who have taken medications during the prior month that could influence trial results (e.g. medications for epilepsy, depression, panic disorders, schizophrenia, etc.), (2) pregnant or breast-feeding women, (3) Korean Medicine Doctors (KMD) or Korean Medicine University students (because we think that they can distinguish between placebo and real acupuncture better than the general population), (4) individuals who drank alcohol or coffee, or overworked the day before or the day of the experiment, and (5) individuals who are determined to be unsuitable for following the study protocol by the researcher.

### Recruitment

We will upload an advertisement to the hospital and university homepages, and place a poster advertisement on the bulletin boards of the hospital and university. Potential participants will call the clinical research coordinator (CRC) and will be pre-screened for eligibility. Eligible participants will make an appointment. When potential participants visit the hospital, the investigator will provide information about volunteering and explain the risks and benefits of the research. When the individuals agree to enroll in the research, they will sign an informed consent form.

### Randomization and allocation concealment

After signing the informed consent form, the participants will be randomized to Group 1 or Group 2. Group 1 will receive the Real Streitberger’s needle (Real ST), Sham Streitberger’s needle (Sham ST), and Phantom acupuncture (PHNT); Group 2 will receive the PSD with a real needle (Real Park), the PSD with a sham needle (Sham Park), and no treatment (No treat). The treatment orders in both groups will be randomized. A computerized balanced block randomization will be performed by an independent clinical research coordinator (CRC) using R version 3.1.2 with ‘block random’ function. The CRC will send an e-mail regarding the name, random number, and treatment order of enrolled participants to the practitioners and operators. This e-mail will be documented in the trial master file (TMF).

### Blinding

Participants will be blinded in this trial. However, it is almost impossible to blind practitioners and operators because they need to know which acupuncture devices to use. They will not be allowed to communicate with the participants concerning the type of acupuncture devices and the purpose of the study. Outcome assessors and statisticians will also be blinded to treatment allocation.

### Intervention

#### Study flow

**Fig. 1:**
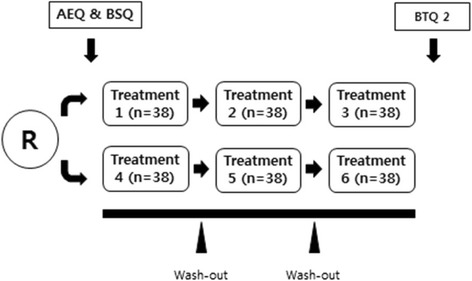
Study flow. *AEQ* indicates acupuncture expectation questionnaire, *BSQ* bodily sensation questionnaire, *BTQ2* blinding test questionnaire 2, *R* randomization

The study flow will be as follows. Group 1 will consist of Sham ST, Real ST, and PHNT. Group 2 will consist of Real Park, Sham Park, and No treatment. Groups 1 and 2 are separate groups; participants allocated to Group 1 will not receive Real Park, Sham Park, and No treatment and vice versa. Participants will have a total of three sessions (acupuncture or no treatment) in a day (Fig. 1). Before acupuncture treatment, the investigator will explain to participants that they will receive three different types of acupuncture except for the no treatment session. Participants will not know that placebo acupuncture occurred until all sessions are completed. To avoid order effect, the order of the three treatment sessions in each group will be randomly assigned by the CRC. Before the first session, the CRC will collect demographic information (e.g., age, sex) and baseline data (e.g., vital signs, acupuncture expectation questionnaire (AEQ) [6], and bodily sensation questionnaire (BSQ)). After each treatment session, participants will complete the blinding test questionnaire 1 (BTQ 1) and acupuncture sensation questionnaire (ASQ) and will be asked about adverse events (AEs). After completion of the third BTQ 1 and ASQ, an independent assessor will inform the participants of the use of placebo acupuncture and instruct them to complete the blinding test questionnaire 2 (BTQ 2).

#### Procedures of each session

**Fig. 2:**
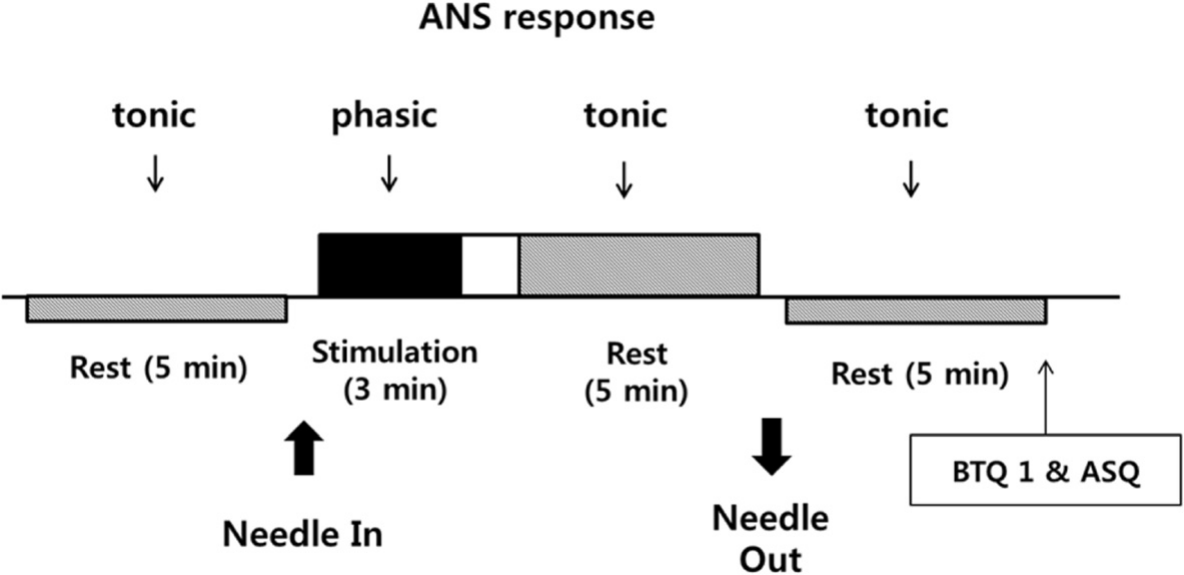
Acupuncture treatment schedule and ANS measurement. *ASQ* indicates acupuncture sensation questionnaire, *BTQ1* blinding test questionnaire1

Physiological data including heart rate (HR) and skin conductance response (SCR) will be measured by an operator during each session to quantify the autonomic nervous system (ANS) response (Fig. 2). We will record three tonic responses (before, during, and after acupuncture treatment) and one phasic response (during stimulation).

1. Stabilization: After all preparations (e.g., attaching electrodes, checking the ECG and SCR wave patterns) are complete, participants will lie on the bed for 5 to10 min to stabilize.
2. Tonic response before acupuncture: We will measure the tonic ANS response before acupuncture treatment during 5 min of resting.
3. Needle insertion and rest: We will insert the acupuncture needle and induce the de-qi sensation for 1 min and explain to participants that the response induced by needle insertion will be measured. To minimize the influence of the acupuncture insertion, participants will be rested for 1 min.
4. Phasic response during stimulation: The practitioner will approach her hand to the left ST 36 acupuncture point and rotate the needle eight times within 3 min according to an audio signal relayed via a headphone. The stimulation timing will be pseudo-randomly set by computer (Psychtoolbox and Matlab, The MathWorks Inc., MA, USA).
5. Rest: To minimize the influence of the acupuncture stimulation, participants will be rested for 1 min.
6. Tonic response during acupuncture: We will measure the tonic ANS response for 5 min during acupuncture treatment.
7. Needle removal and rest: We will remove the acupuncture needle for 1 min. To minimize the influence of the acupuncture needle removal, participants will be rested for 1 min.
8. Tonic response after acupuncture: We will measure the tonic ANS response for 5 min after the acupuncture needle is removed.

#### Acupuncture treatment setting and equipment

**Fig. 3:**
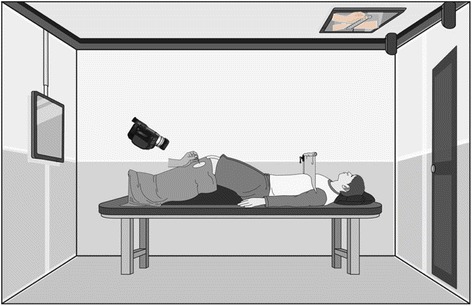
Acupuncture treatment setting

The acupuncture treatment room setting will be modified from a previous study by Lee et al. [5] and is shown in Fig. 3. Participants will lie on the bed in a supine position. A visual barrier will prevent participants from viewing their leg and the acupuncture treatment procedure directly.

Acupuncture stimulation will be recorded simultaneously and will be shown to the participants through a ceiling monitor in real time for 3 min. Before the stabilization phase, the operator will show the acupuncture point and movement of their legs through the ceiling monitor so that participants will have body ownership via the image.

Details of the acupuncture treatment plans are shown in Table 1.

**Table 1:** Details of acupuncture treatments (STRICTA 2010 checklist)

Item		Detail
1. Acupuncture rationale	1a) Style of acupuncture	Manual acupuncture based on traditional Korean medicine theory
	1b)Reasoning for treatment provided, based on historical context, literature sources, and/or consensus methods, with references where appropriate	Consensus of the KMD
	1c)Extent to which treatment was varied	All participants will receive standardized treatment
2. Details of needling	2a) Number of needle insertions per subject per session	1
	2b) Names of points used	Left ST36
	2c) Depth of insertion, based on a specified unit of measurement	15 mm
	2d) Response sought	De-qi
	2e) Needle stimulation :	During 3 min of phasic session, manual stimulation will be conducted eight times based on an audio signal received via a headphone. Interval between each manual stimulation is from 10 to 14 s. For each manual stimulation, the acupuncture needle will be rotated five times during 3 s.
	2f) Needle retention time	11 min
	2g) Needle type	[Group 1] Real Streitberger’s needle; 0.25 × 40 mm sterilized stainless steel needle (Asia-med GmbH & Co. KG, Kirchplatz 1, Germany).
		[Group 2] PSD; 0.25 × 40 mm sterilized stainless steel needle (Park sham Device, Acuprime, Exter, UK).
3. Treatment regimen	3a) Number of treatment sessions	3 sessions
	3b) Frequency and duration of treatment sessions	3 sessions for 1 day
		(30 min washout period between each session)
4. Other components of treatment	4a) Details of other interventions administered to the acupuncture group	None
	4b) Setting and context of treatment, including instructions to practitioners, and information and explanations to patients	The study will be conducted in the Kyung Hee University Medical Center. All information except the objective of trial and acupuncture types will be provided to the participants.
5. Practitioner background	5) Description of participating acupuncturists	Licensed KMD with at least 5 years of clinical practice experience
6. Control interventions	6a) Rationale for the control or comparator in the context of the research question, with sources that justify this choice	Validated placebo acupuncture devices will be used in control sessions.
	6b) Precise description of the control or comparator. If sham acupuncture or any other type of acupuncture-like control is used, provide details as for Items 1 to 3 above.	The order of three treatments in each group will be randomized.
		[GROUP 1] 1) Sham Streitberger’ needle: 0.25 × 40 mm sterilized stainless steel needle (Special No. 16 of Asia-med GmbH & Co. KG, Kirchplatz 1,Germany) will be used. The appearance, acupuncture point, stimulation method, and duration will be the same with the real Streitberger’s needle.
		2) Phantom acupuncture: There is no tactile stimulation to ST 36. To induce credibility that participants are treated with acupuncture, the video clip of the previously recorded Streitberger’s needle session will be shown.
		[GROUP 2] 1) PSD with sham needle: 0.25 × 40 mm sterilized stainless steel needle (Park sham Device, Acuprime, Exter, UK) will be used. The appearance, acupuncture point, stimulation method, and depth will be the same with the PSD as with the real needle. 2) No treatment: There is no acupuncture treatment. To induce the attention effect as in the other sessions, a neutral image will be provided instead of an acupuncture treatment image.

*KMD* Korean Medicine Doctor; *PSD* park sham device

#### Phantom acupuncture and no treatment session

In PHNT, practitioners will not provide any tactile stimulation and will only approach their hand to the left ST 36 acupuncture point without touching. A recorded video clip of the participant’s Real ST session will be played during the PHNT session. If the PHNT session is scheduled as the first session, we will play a video from another participant with similar skin color and leg hair.

In the No treatment session, practitioners will inform participants that they will not receive any treatment. A blinding questionnaire will not be completed; recorded physiological data will be used as a baseline. In order to ensure comparability, participants will undergo the same procedure except they will be shown a 3 min video clip that is not related to acupuncture but has a similar background color and shows some type of artificial movement that would occur with same stimulation time point during an acupuncture session.

### Outcome measurement

#### Primary endpoint

BTQ 1 : After each acupuncture session, participants will complete the BTQ 1. BTQ1 consists of five questions. If a participant completes all five questions without any doubt or asking, we will conclude blinding is succeeded. If participants mention strange aspect of the questionnaire or reply they did not receive acupuncture treatment, conclude that blinding is failed. Otherwise, we will conclude that blinding is succeeded.

#### Secondary endpoints

##### Bang’s blinding index (BI)

After all the sessions are finished, participants will complete the BTQ 2. At this time, participants will be told that a placebo acupuncture session occurred. We will calculate the BI from the BTQ 2 [7]. The range of BI is −100 to 100 %; 0 % means random guessing (e.g., 50 % correct and 50 % incorrect) and 100 % means complete unblinding (i.e., all responses are correct), −100 % means opposite of guessing (e.g., all responses are incorrect) [8]. Thus, the BI is the proportion of participants who guessed their treatment arm correctly beyond chance [9, 10].

##### Massachusetts General Hospital (MGH) Acupuncture Sensation Scale (MASS) Index by de-qi sensation questionnaire (DSQ)

The MASS measures the sensation caused by acupuncture stimulation. It consists of 12 descriptions: soreness, aching, deep pressure, heaviness, fullness/distention, tingling, numbness, sharp pain, dull pain, warmth, cold, and throbbing. The MASS index is a weighted average based on a formula [11]. We will determine the MASS using the DSQ.

##### Heart rate, heart rate variability, and skin conductance response

Participants will rest for 5 to10 min before we measure their tonic response to stabilize the electrocardiogram and SCR wave pattern. We will record the HR and SCR throughout the study using a PowerLab Data acquisition system/800, Bio amplifier/ML132, GSR Amplifier/ML116 (AD instruments, Australia) with a 400 Hz sampling rate. To measure HR, participants will be placed on the bed and three electrodes (3M disposable PAD 2223) will be attached beneath the clavicles and the left rib. Then, we will remove power noise frequency at 60 Hz and detect the R peak to measure the HR. To measure the SCR, we will apply a gel to the index and third fingers and attach electrodes [5, 12].

The HR and SCR will be measured during the tonic and phasic responses. For the tonic responses, the mean HR and SCR will be measured before, during, and after the acupuncture treatment session for 5 min.

The HRV will be calculated by evaluating the R peak. In the time domain analysis, we will calculate the standard deviation of all R-R intervals (SDNN) and the root-mean-square of the successive difference (RMS-SD). For the frequency domain analysis, we will calculate the high frequency value (HF), low frequency value (LF), total power, and the LF/HF ratio [13, 14].

In the phasic event related SCR responses, the average of the maximum score change and the area under the curve (AUC) will be estimated. We will also calculate the stimulation/baseline SCR ratio of the score change and the AUC.

### Sample size calculation

This will be a kind of mechanism study, so we did not follow the general method to calculate sample size. It is known that a sample size of 12 or more is adequate for a pilot study [15, 16]. We concluded that we will have sufficient analytical power if our sample size is greater than 12. We decided that we need to enroll at least 30 participants in our pilot study for each session assuming a drop-out rate of approximately 20 %. We calculated a sample size of 37.5 and rounded to this to 38 resulting in a total sample size of 76 participants.

### Statistical analysis

As our design has two independent groups that acquire comparability by randomization, we can compare variables not only within each group, but also between groups.

The primary outcome of our research will be to compare the blinding rates of the placebo acupuncture devices studied within and between groups. We will also conduct several exploratory secondary outcome analyses. Expectation for acupuncture (measured by AEQ) [6], previous acupuncture experiences, and age will be considered as covariates. We will analyze physiological data in three tonic responses (before, during, and after acupuncture) and during phasic responses. We will not statistically compare the Bang’s BI [9]. Instead, we will calculate the point estimate and 95 % confidence interval of Bang’s BI.

For dichotomous variables (i.e., blinding rate), within group comparison will be performed using Cochran’s Q test for Group 1 (Sham ST, Real ST, and PHNT) and McNemar’s test for Group 2 (Sham, Real Park). We will not check the blinding of the No treatment session. Comparisons of blinding rates between group devices (Sham ST vs Sham Park or PHNT vs Sham Park) will be performed using the chi-square test, and comparisons within group devices (PHNT vs Sham ST) will be performed using McNemar’s test. For continuous variables (i.e., the MASS index, De-qi sensation, physiological data such as HR, HRV, and SCR), within group comparisons will be performed using repeated measured ANOVA for Group 1 and paired *t*-test for Group 2. We will use the independent *t* test for comparisons between group.

When we analyze physiological data, we will use the both change ratio contrast to baseline and absolute value. Change ratio and absolute value of HR and SCR will be compared for three separate windows during the tonic responses (before vs during vs after acupuncture session). As absolute change value is very dependent on baseline value, it will be used as covariates.

### Quality control

Before the study begins, we will conduct several simulations using our colleagues and volunteers to identify any problems with our study protocol. Practitioners will conduct acupuncture sessions according to the standard operating procedure (SOP) of our study. We will have researcher meetings regularly to discuss issues that may be raised by investigators and participants such as protocol revisions, serious adverse events, and participant recruitment.

### Safety and adverse event outcomes

When each session is completed, the assessor will ask if any AE occurred and it will be documented in the CRF. If any AEs occur, the type of AE, start/end date, severity, manner of report, course, outcome, causality with acupuncture treatment, and actions taken will be documented and appropriate treatment will be provided to the participants.

### Ethics approval and registration

This study has been approved by the Institutional Review Board of Kyung Hee University Korean Medicine Hospital (KOMCIRB-140923-HR-008). Written informed consent will be obtained from all participants. This study has been registered with the Clinical Research Information Service (CRIS), Republic of Korea, KCT0001347 (registered: January 15th, 2015).

## Discussion

This trial was designed to investigate if various placebo acupuncture devices are appropriate for control groups in acupuncture research. We will evaluate the blinding of participants and also measure physiological parameters such as HR, HRV, and SCR. In addition, we will investigate the effects of various factors on blinding and physiological parameters. We will examine the effects of the bodily sensation, de-qi sensation (i.e., acupuncture-evoked sensations including numbness, heaviness, soreness, or distention), or patient expectation for acupuncture on blinding and physiological parameters.

Acupuncture treatment is composed of a variety of components such as acupuncture point selection, skin penetration, stimulation dose (diameter, length, and number of needles, needling depth, and stimulation method), patient expectation, and practitioner-patient relationship [2]. However, studies on how each factor affects the total therapeutic effect are lacking. In this study, we are going to dis-associate a range of acupuncture treatment factors using a validation test of three types of placebo acupuncture techniques that have different components.

Other researchers have developed placebo acupuncture devices and evaluated their relevance using validation tests.

Streitberger et al. performed a validation study that investigated if acupuncture- naïve healthy participants (*n* = 60) could feel the difference between a real acupuncture needle and a placebo acupuncture device [3]. They showed that the participants could not distinguish between the two needles and suggested Streitberger’s needle as a credible placebo treatment. Also White et al. conducted a validation test of Streitberger’s needle in participants waiting for orthopedic hip and knee joint replacement [17]. Most participants could not distinguish penetration with a real acupuncture needle from that with a placebo needle. However, in two similar treatments, almost 40 % were able to discern a difference between real and placebo acupuncture treatment administered by the male practitioner only. This shows that it is important how two interventions are delivered and that standardization of treatment technique is strictly required.

The PSD developed by Park et al. was validated in two randomized controlled trials (RCTs) in acute stroke patients and acupuncture-naïve, healthy volunteers [18]. The study was designed to evaluate if PSD was indistinguishable from a real acupuncture needle and if PSD induced de-qi. They found that PSD was indistinguishable and inactive (in terms of de-qi), thus it is a valid placebo control for acupuncture research.

However, these previous two devices touch the skin and thus induce a physiological effect. To overcome this limitation, Lee et al. developed a phantom acupuncture technique that imitates the acupuncture treatment ritual without any tactile stimulation [5]. They showed the credibility of phantom acupuncture without somatosensory components of real acupuncture in healthy participants.

In summary, three types of placebo acupunctures were all evaluated to blinded participants using validation tests. In this study, by measuring blinding, de-qi sensation, and various physiological parameters such as HR, HRV, and SCR, we will investigate if placebo acupuncture techniques are indistinguishable from real acupuncture and physiologically inert.

This trial will evaluate the relevance of using placebo acupuncture devices in control groups using a validation test of various placebo acupuncture devices that have been used in acupuncture research.

### Trial status

This trial is currently recruiting participants.

## Abbreviations

AEQ: acupuncture expectation questionnaire
ANS: quantify autonomic nervous system
ASQ: acupuncture sensation questionnaire
BTQ: blinding test questionnaire
BSQ: bodily sensation questionnaire
BI: blinding index
CRC: clinical research coordinator
CRF: case report form
DSQ: de-qi sensation questionnaire
HRV: heart rate variability
IRB: Institutional Review Board
KMD: Korean Medicine Doctor
MASS Index: Massachusetts General Hospital Acupuncture Sensation Scale index
No treat: no treatment session
PHNT: phantom acupuncture
PSD: park sham device
Real Park: park sham device with real needle
Real ST: Real Streitberger’s needle treatment
Sham Park: park sham device with sham needle
Sham ST: Sham Streitberger’s needle treatment
SCR: skin conductance response
SOP: standard operation procedures
TMF: trial master file

## References

[1] NIH Consensus Conference (1998). Acupuncture. JAMA.

[2] Zhu D, Gao Y, Chang J, Kong J (2013). Placebo acupuncture devices: considerations for acupuncture research. Evid-Based Complement Altern Med ECAM.

[3] Streitberger K, Kleinhenz J (1998). Introducing a placebo needle into acupuncture research. Lancet.

[4] Park J, White A, Lee H, Ernst E (1999). Development of a new sham needle. Acupunct Med.

[5] Lee J, Napadow V, Kim J, Lee S, Choi W, Kaptchuk TJ (2014). Phantom acupuncture: dissociating somatosensory and cognitive/affective components of acupuncture stimulation with a novel form of placebo acupuncture. PLoS One.

[6] Dennehy EB, Webb A, Suppes T (2002). Assessment of beliefs in the effectiveness of acupuncture for treatment of psychiatric symptoms. J Altern Complement Med N Y N.

[7] Bang H, Ni L, Davis CE (2004). Assessment of blinding in clinical trials. Control Clin Trials.

[8] Bang H, Flaherty SP, Kolahi J, Park J (2010). Blinding assessment in clinical trials: a review of statistical methods and a proposal of blinding assessment protocol. Clin Res Regul Aff.

[9] Lee H, Bang H, Kim Y, Park J, Lee S, Lee H (2011). Non-penetrating sham needle, is it an adequate sham control in acupuncture research?. Complement Ther Med.

[10] Kolahi J, Bang H, Park J (2009). Towards a proposal for assessment of blinding success in clinical trials: up-to-date review. Community Dent Oral Epidemiol.

[11] Kong J, Gollub R, Huang T, Polich G, Napadow V, Hui K (2007). Acupuncture de qi, from qualitative history to quantitative measurement. J Altern Complement Med N Y N.

[12] Chang D-S, Kim Y-J, Lee S-H, Lee H, Lee I-S, Park H-J (2013). Modifying bodily self-awareness during acupuncture needle stimulation using the rubber hand illusion. Evid-Based Complement Altern Med ECAM.

[13] Chung JWY, Yan VCM, Zhang H (2014). Effect of acupuncture on heart rate variability: a systematic review. Evid Based Complement Alternat Med.

[14] Evrengül H, Tanriverdi H, Dursunoglu D, Kaftan A, Kuru O, Unlu U (2005). Time and frequency domain analyses of heart rate variability in patients with epilepsy. Epilepsy Res.

[15] Hertzog MA (2008). Considerations in determining sample size for pilot studies. Res Nurs Health.

[16] Julious SA (2005). Sample size of 12 per group rule of thumb for a pilot study. Pharm Stat.

[17] White P, Lewith G, Hopwood V, Prescott P (2003). The placebo needle, is it a valid and convincing placebo for use in acupuncture trials? A randomised, single-blind, cross-over pilot trial. Pain.

[18] Park J, White A, Stevinson C, Ernst E, James M (2002). Validating a new non-penetrating sham acupuncture device: two randomised controlled trials. Acupunct Med J Br Med Acupunct Soc.

